# A Review on Non-invasive Respiratory Support for Management of Respiratory Distress in Extremely Preterm Infants

**DOI:** 10.3389/fped.2020.00270

**Published:** 2020-05-28

**Authors:** Yuan Shi, Hemananda Muniraman, Manoj Biniwale, Rangasamy Ramanathan

**Affiliations:** ^1^Ministry of Education Key Laboratory of Child Development and Disorders, Key Laboratory of Pediatrics, Children's Hospital of Chongqing Medical University, Chongqing, China; ^2^Department of Pediatrics, Creighton School of Medicine, Omaha, NE, United States; ^3^Neonatology Association Limited, Obstetrix Medical Group of Phoenix, Mednax, Arizona, AZ, United States; ^4^Division of Neonatology, LAC+USC Medical Center, Keck School of Medicine of the University of Southern California, Los Angeles, CA, United States

**Keywords:** bronchopulmonary dysplasia (BPD), nasal continuous positive airway pressure (NCPAP), nasal intermittent positive pressure ventilation (NIPPV), high flow nasal cannula (HFNC), nasal high frequency ventilation (NHFV), noninvasive ventilation (NIV), noninvasive ventilation-neurally adjusted ventilatory assist (NIVNAVA)

## Abstract

Majority of extremely preterm infants require positive pressure ventilatory support at the time of delivery or during the transitional period. Most of these infants present with respiratory distress (RD) and continue to require significant respiratory support in the neonatal intensive care unit (NICU). Bronchopulmonary dysplasia (BPD) remains as one of the major morbidities among survivors of the extremely preterm infants. BPD is associated with long-term adverse pulmonary and neurological outcomes. Invasive mechanical ventilation (IMV) and supplemental oxygen are two major risk factors for the development of BPD. Non-invasive ventilation (NIV) has been shown to decrease the need for IMV and reduce the risk of BPD when compared to IMV. This article reviews respiratory management with current NIV support strategies in extremely preterm infants both in delivery room as well as in the NICU and discusses the evidence to support commonly used NIV modes including nasal continuous positive airway pressure (NCPAP), nasal intermittent positive pressure ventilation (NIPPV), bi-level positive pressure (BI-PAP), high flow nasal cannula (HFNC), and newer NIV strategies currently being studied including, nasal high frequency ventilation (NHFV) and non-invasive neutrally adjusted ventilatory assist (NIV-NAVA). Randomized, clinical trials have shown that early NIPPV is superior to NCPAP to decrease the need for intubation and IMV in preterm infants with RD. It is also important to understand that selection of the device used to deliver NIPPV has a significant impact on its success. Ventilator generated NIPPV results in significantly lower rates of extubation failures when compared to Bi-PAP. Future studies should address synchronized NIPPV including NIV-NAVA and early rescue use of NHFV in the respiratory management of extremely preterm infants.

## Introduction

Providing optimal ventilation strategies remains the key to success of managing extremely preterm infants. Majority of the extremely preterm infants have respiratory distress (RD) needing significant respiratory support immediately after birth or after admission to the neonatal intensive care unit (NICU) due to poor inspiratory effort, weak intercostal muscles, and poor diaphragmatic function. These infants are at very high risk of developing bronchopulmonary dysplasia (BPD) and adverse neurodevelopmental outcomes, which are directly related to the duration of invasive mechanical ventilation (IMV) and supplemental oxygen. The strong association between ventilator dependency and neurologic injury, such as severe intraventricular hemorrhage (IVH) and periventricular leucomalacia, emphasizes the severity of their illness (1). A recent study showed more than 60 days of positive pressure support regardless of invasive or non-invasive ventilation (NIV) mode was associated with a higher risk for neurodevelopmental problems (2). Avoiding intubation and using NIV modes in preterm infants minimizes the risk for lung injury and optimizes neonatal outcomes. Use of nasal continuous positive airway pressure (NCPAP) in the delivery room and nasal intermittent positive pressure ventilation (NIPPV) in the NICU has been shown to decrease the need for IMV in extremely preterm infants without increasing major morbidities (3).

In extremely preterm infants, BPD is associated with long term impaired pulmonary function and adverse neurological outcomes (4–6). While the etiology of BPD is multifactorial, lung injury particularly with IMV and resulting inflammation play a major role in the pathogenesis (7). NIV has been shown to reduce the risk of BPD when compared to IMV (8). Ventilatory practices have evolved over the last few decades with preference for NIV in the management of respiratory distress syndrome (RDS) in extremely preterm infants (4). Also, the definition of BPD is constantly evolving. Existing definitions mostly relied on level and duration of supplemental oxygen and did not take into account the major changes in NIV modes that are currently used in preterm infants. Recently, in a study using 18 pre-specified definitions of BPD that used disease severity based on level of respiratory support and supplemental oxygen at 36 weeks' postmenstrual age (PMA), only mode of respiratory support best predicted early childhood morbidity, regardless of supplemental oxygen use. Our focus should be to assess and follow the extremely preterm infants based on level of non-invasive as well as invasive positive pressure support at the time of discharge and after discharge (9).

## Modes of Non-Invasive Ventilation

**Figure 1:**
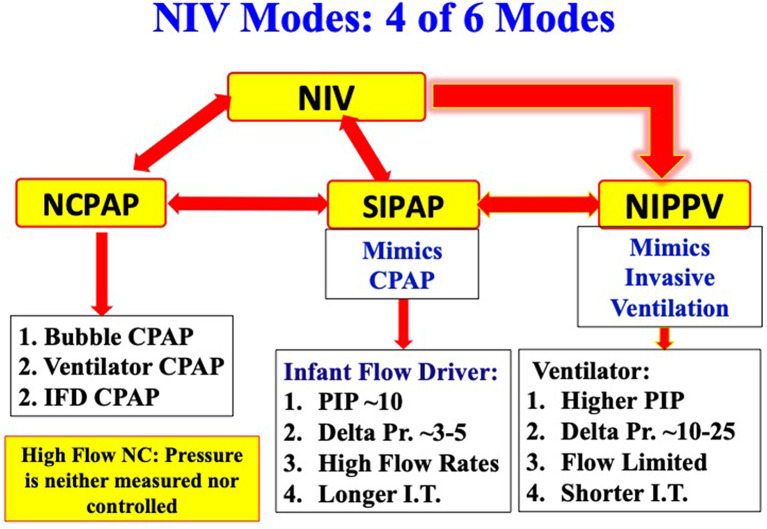
Common modes of noninvasive ventilation.

**Figure 2:**
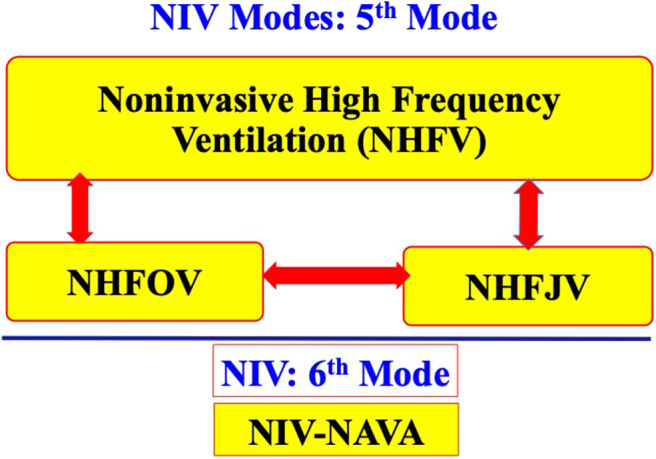
Newer modes of NIV.

**Figure 3:**
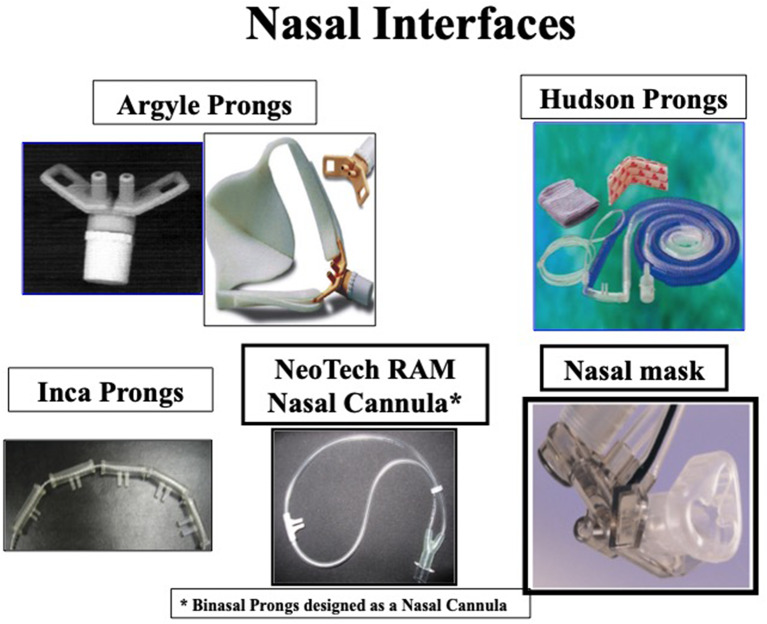
Nasal interfaces to provide NIV.

Six modes of NIV are currently used in extremely preterm infants. Four of the six modes commonly used in most of the NICUs include NCPAP, bilevel positive airway pressure (Bi-PAP) or sigh breaths above a baseline CPAP pressure (Si-PAP), NIPPV and high flow nasal cannula (HFNC) (Figure 1). Remaining 2 modes, namely, nasal high frequency ventilation (NHFV) using nasal high frequency oscillatory ventilation (NHFOV) or nasal high frequency jet ventilation (NHFJV) and non-invasive neurally adjusted ventilatory assist (NIV-NAVA) are not well-studied in extremely preterm infants and need further evaluations before routine use of these modes (Figure 2). Positive pressure delivery during NIV involves pressure generators, nasal interfaces (Figure 3) and ability to provide one or two levels of pressures at different ventilator rates. NIV in the NICU can be provided by these modalities either as a primary mode of respiratory support or following extubation after a period of IMV. NHFV modes are also being used increasingly as a rescue mode to treat hypercarbia and to decrease the need for intubation in extremely preterm infants failing other modes of NIV support. NIV can also be used in combination with early, rescue surfactant treatment. Surfactant can be delivered using invasive techniques such as INSURE (INtubation, SURfactant, and Extubation), minimally invasive techniques including SurE (surfactant without endotracheal intubation) using a feeding tube or a specially designed catheter, laryngeal mask airway, or non-invasive technique, like, nebulization (10–13).

## Mechanisms of NIV

Physiological mechanisms leading to improvement in the lung mechanics are somewhat similar with all these modes of NIV. NCPAP reduces upper airway resistance, helps to establish functional residual capacity (FRC), decreases chest wall distortion, augments spontaneous breathing efforts, preserves endogenous surfactant, decreases the need for surfactant administration, and decreases the need for, and duration of IMV (14). However, in patients with hypopnea or apnea, NCPAP often fails, needing intubation and IMV. Providing a backup rate using NIPPV with adequate peak inspiratory pressure (PIP) decreases apneic spells, improves ventilation and decreases the need for intubation. NIPPV is a time cycled, pressure limited mode of ventilation. Conventional ventilator is used to generate two levels of pressures, namely, PIP and positive end expiratory pressure (PEEP). Additionally, a backup rate is provided typically using longer inspiratory time. Benefits of NIPPV mode include all of the benefits of NCPAP listed above, and pharyngeal dilation with further decrease in upper airway resistance, augmentation of spontaneous inspiratory effort via Head's paradoxical reflex, improving compliance and reopening of partially collapsed airways, increase in FRC, increase in tidal volume (Vt) and minute volume, better alveolar recruitment due to higher mean airway pressure (MAP), reduction in chest wall distortion, and improved respiratory unloading with decrease in work of breathing (15). Head's paradoxical reflex is seen typically during a rapid inflation of the lungs causing a deep inspiration or gasp. It is mediated by the irritant receptors of the major airways receptive to lung inflation. The reflex is seen most commonly on the first day and may help to establish and maintain FRC. Head's paradoxical reflex has also shown to possibly increase neural inspiratory time in patients receiving NIV-NAVA (16). NIPPV also stimulates the Hering-Breuer inflation reflex with inflation of lungs resulting in cessation of respiratory activity preventing hyperinflation. This reflex is mediated through the stretch receptors in the smooth muscles of the major airways and is time-dependent with a longer inspiratory time resulting in a longer period of respiratory inhibition before the next breath. Both NCPAP and NIPPV modes may trigger this reflex, causing slower spontaneous respiratory rate. In the preterm infants, this reflex produces rapid, shallow tidal breathing. In older infants this reflex prevents excessive tidal volumes and can only be stimulated if the inflating volume is increased beyond a critical threshold.

Proposed mechanisms for the use of HFNC include washout of nasopharyngeal dead space, decrease in inspiratory resistance, and provision of positive pressure. However, pressure generated during HFNC is neither measured nor controlled by the clinician and is very unpredictable (17). NHFV modes promote better lung recruitment and removal of carbon dioxide.

## Devices for Delivering NIV

NCPAP may be provided using a water column as a resistor to generate CPAP (Bubble CPAP), or using a flow generator as in infant flow driver (IFD) device, or using a conventional ventilator with continuous or variable flow rates. Studies comparing different modes of providing NCPAP have shown no significant difference in extubation failure rates (18, 19). Most commonly used bi-level modes include Bi-PAP, Si-PAP, and Duo-PAP. IFDs are variable flow devices, and generate two levels of pressures, a high pressure and a low pressure or CPAP by varying the flow rates. Bi-PAP/Si-PAP mimics NCPAP due to low delta pressure in these modes. The delta pressure during bi-level mode is between 5 and 10 cmH_2_O, which often is not enough to treat hypercapnia or support poor spontaneous respiratory efforts.

Most of the conventional ventilators can provide NIPPV with PIP, PEEP, and rate to provide adequate support. Furthermore, flow can be adjusted to provide adequate pressure or compensate for leaks. Newer ventilators have NIV modes with excellent leak compensation. Only one type of ventilator provides NIV-NAVA mode. Both high frequency oscillatory ventilator as well as high frequency jet ventilators can be used to provide NHFV. HFNC is provided by dedicated devices where only flow is adjusted to optimize gas exchange.

## Nasal Interfaces in Delivery Room and NICU

Common interfaces used in the delivery room (DR) to provide NIV support are round and anatomical mask, single or bi-nasal nasopharyngeal prongs, bi-nasal prongs, nasal mask, or RAM nasal cannula (NC) (Neotech RAM Nasal Cannula®, Neotech Products, Valencia, California, USA) with pressure generating devices including self-inflating or flow-inflating bag, and T piece resuscitator (20). Bag and mask resuscitation is often not effective in the DR even when performed by the experienced personnel in extremely preterm infants. Three major issues with bag and mask ventilation are: mask leak, upper airway obstruction from the tongue falling backwards toward the oropharynx and increase in dead space with the gas in the oropharynx not contributing to gas exchange (21–24). Corrective ventilation steps during resuscitation are taught using MRSOPA mnemonic: Mask adjustment, Repositioning airway, Suctioning, Opening the mouth, Increasing inspiratory pressure, and Alternative airway. These steps are not always successful, especially among very preterm infants. The greater the number of MRSOPA steps used in the DR, the more likely intubation occurred (25). In another study, MRSOPA maneuvers improved tidal volume delivery in some cases, but, worsened exhaled tidal volumes in others. In fact, these authors found MRSOPA steps actually induced mask leak and airway obstruction in some cases (26). In a randomized, controlled trial, use of NC vs. face mask for primary neonatal resuscitation in the DR in more mature neonates (mean GA 36 weeks), NC use resulted in significantly less need for intubation (0.6 vs. 6.3%; *p* < 0.001) and chest compressions (1.65 vs. 8.28%; *p* = 0.001) in the NC group (27).

**Figure 4:**
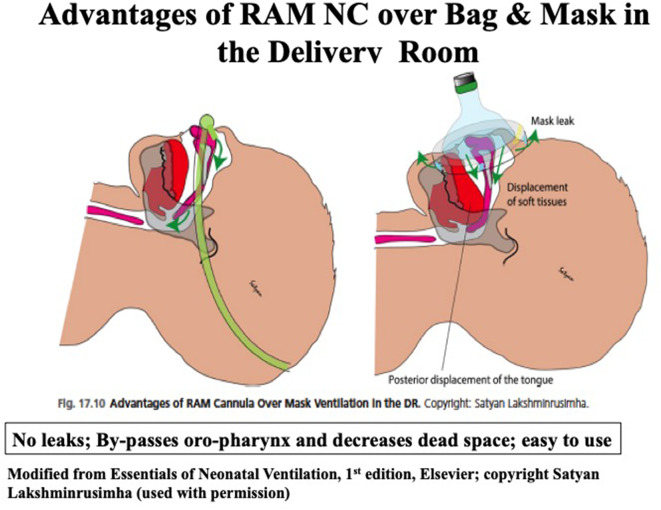
Application of RAM NC in the NICU.

Successful use of RAM NC for the resuscitation of very low birth weight infants and decreased the need for intubation even among the lower gestational age infants (mean GA 27 weeks) has been reported (28). A recent study in <29 weeks' gestation infants used sustained lung inflation (SLI) followed by NCPAP ranging from 6 to 8 cmH_2_O, using RAM NC resulted in a significant reduction in intubation rates in the DR (29). Success with the use of RAM NC as an interface is likely due to delivery of tidal volume through the nasopharynx, eliminating dead space by avoiding oropharyngeal space, ease of application with T-Piece resuscitator, and minimizing upper airway obstruction (Figure 4) (30). In addition, sustained inflation or NIPPV may be applied in preterm infants needing additional support without manipulating the tiny infants during resuscitation. Among the pressure delivery devices, T piece resuscitator delivers targeted inflation pressure more consistently compared to self-inflating or flow inflating bag although this has not been shown to improve clinical outcomes (31).

**Figure 5:**
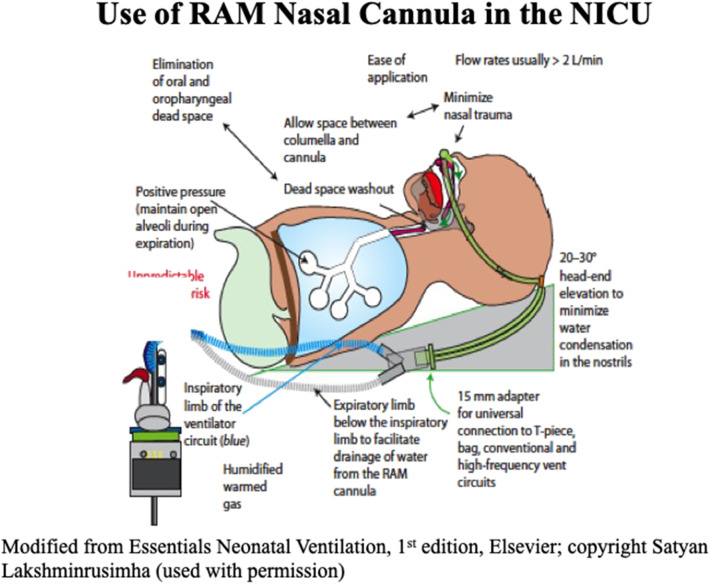
NCPAP in delivery room and early failures.

Various interfaces used in the NICU include varieties of short nasal prongs, RAM NC and nasal masks. There is considerable variation in measured resistance between these interfaces. When applying smallest size interfaces for extremely preterm infants all nasal interfaces result in decrease in pressures due to high resistance. Pressure drop may vary based on the set flow, internal diameter and length of the prongs. Use of interfaces with high resistance may result in a greater drop in delivered airway pressure in comparison to set pressure (32). Application and advantages of RAM NC to provide NIV in the NICU are shown in Figure 5. Clinicians need to be aware of adjusting pressure and flow settings while using the ventilator to provide NIV. A meta-analysis of studies with nasal mask comparing to binasal prongs showed significantly decreased the risk of CPAP failure (4 RCTs [*N* = 459]; relative risk [RR]: 0.63; 95% confidence interval [CI]: 0.45–0.88; *P* = 0.007; *I*^2^ = 0%, NNT: 9), and the incidence of moderate to severe nasal trauma (3 RCTs [*N* = 275], RR: 0.41; 95%CI, 0.24–0.72; *P* = 0.002; *I*^2^ = 74%, NNT: 6) (33). Larger studies are needed to validate safety and efficacy of using nasal masks in extremely preterm infants.

### Pressure Transmission During NIPPV

Pressure transmission to the hypopharynx or to the lung during NIPPV is difficult to measure. It depends on the size of the prongs, length of tubing, leaks around the nostrils, and set inspiratory time and whether the infant's mouth is open or closed. Using a computerized test lung simulator, pressure transmission using different size RAM NCs has been reported. In this well-designed study with a 30% leak, authors demonstrated around 70, 80, and 90% of set PIP delivered to the hypopharynx with preemie, newborn, and infant size RAM NCs respectively (34). More studies are needed comparing similar diameter prongs and similar leak settings. If a higher CPAP or PEEP is needed, then, the leak at the nasal interface may be decreased by using cannulaide® (Beever Medical Solutions, OR, USA).

### Temperature and Humidity During NIV

Heating and humidification of inspired gas is a routine practice when providing respiratory support in infants. Unconditioned dry and cold gas can result in impaired ciliary function, reduced clearance of secretions, damage to the airway mucosa which may impair lung function by reducing compliance and FRC. Different NIV interfaces deliver inspiratory gases of variable temperature and humidity. Some HFNC and variable flow CPAP devices at higher gas flow may not achieve the recommended temperature and humidity (35). Higher NHFOV settings with low frequencies, high amplitudes, and high inspiratory to expiratory ratios may also place infants at an increased risk of upper airway injury due to decreased humidification (36). There are no studies on humidity or temperature of gas delivered in the pharynx with RAM NC or other binasal prongs. Humidification is evidenced by condensation in the tubing of RAM NC. Temperature measurement is done at the wye and beyond that, the tubing length is 11 cm to the prongs. With continuous flow of heated and humidified gas, a significant drop in temperature between the wye and the patient's nasal interface is not expected. Inspiratory gas also gets heated and humidified by the patient's nasopharynx.

## NIV for Stabilization in the Delivery Room

Establishment of FRC during the isovolumic transformation of a fluid filled lung to an air breathing lung is critical for successful adaptation and post-natal transition (20). Extremely preterm infants are at high risk of respiratory distress and maladaptation owing to immature lungs, insufficient production of surfactant, highly compliant chest wall and immature respiratory center control (37). As a result, more than 70% of extremely preterm infants require positive pressure support (38). Strategies to optimize lung recruitment and establishing FRC at this crucial period can play a major role in decreasing respiratory morbidities and mortality in this vulnerable population. Until early 2000s, elective intubation with prophylactic administration of surfactant was the standard of care in the initial management of extremely preterm infants. After large clinical trials showing benefits of NCPAP use in the DR to decrease need for intubation and IMV (39, 40) use of NIV during stabilization and initial treatment of respiratory distress has significantly increased (4).

## NCPAP in Delivery Room

**Figure 6:**
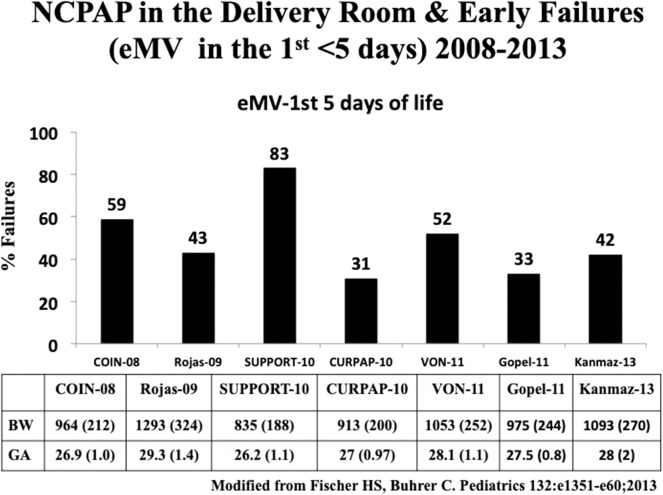
Advantages of RAM NC over Bag & Mask in the delivery room.

CPAP has been shown to be effective in establishing FRC (37). Two large randomized controlled trials (RCT) compared NCPAP with routine intubation in the delivery room (39, 40). The CPAP or intubation at birth (COIN) trial randomized 610 spontaneously breathing infants born at 25 to 28 weeks' gestation with signs of respiratory distress at 5 min of life to receive either CPAP or endotracheal intubation. Infants intubated due to respiratory distress before 5 min of age were excluded. NCPAP of 8 cmH_2_O was used in this study. There was no difference in the primary outcome, namely, death or BPD between the two groups; however, there was a higher incidence of pneumothorax in the CPAP group^.^(39). The surfactant positive airway pressure and pulse oximetry (SUPPORT) trial from USA randomized 1,316 infants between 24 and 28 weeks' gestational age to receive NCPAP or endotracheal intubation along with administration of surfactant. Overall mortality (47.8 and 51%, respectively) and BPD rates were similar between the NCPAP and the intubation with surfactant group (40). Evidence from these studies showed that NCPAP was as effective as routine intubation in the extremely preterm infants. To date, seven RCTs using NCPAP in the delivery room have been published. Failure rates needing intubation and IMV ranged from 31 to 83% (Figure 6; Table 1) (39–45, 47). None of individual clinical trials showed benefit in the primary outcome, namely, death or BPD. However, systematic review and meta-analysis of these studies showed a small but significant benefit in decreasing death or BPD, with a number needed to treat (NNT) of 25 (48). In a long term follow up study by Doyle et al. despite substantial increase in the use of NCPAP, there was no decrease in BPD and more importantly, no improvement in lung function was seen at 8 years of age (49). Exact reasons for the lack of benefit with NCPAP are not clear. It may be due to inability to recruit lungs with inadequate CPAP pressures due to leaks with nasal interfaces or lack of augmentation of breaths as provided during NIPPV.

**Table 1:** Studies comparing NCPAP and intubation with IMV.

**References**	**Intervention (*n*)**	**GA, weeks**	**BPD (%)**	**Death (%)**	**Combined BPD and death (%)**	**Intubation rates in NCPAP group (%)**
Morley et al. (39)	NCPAP (307) vs. IMV (303)	25–28	29 vs. 35	6.5 vs. 5.9	34 vs. 39	59
SUPPORT et al. (40)	NCPAP (663) vs. IMV (653)	24–28	40 vs. 44	14 vs. 17	49 vs. 54	83
Dunn et al. (41)	NCPAP (223) vs. INSURE (216) vs. IMV (209)	26–29	n/a	4 vs. 7 vs 7	30 vs. 28 vs. 36	52
Rojas et al. (42)	NCPAP (137) vs. INSURE (141)	27–30	59 vs. 49	9 vs. 9	62 vs. 54	53
Sandri et al. (43)	NCPAP (105) vs. INSURE (103)	25–29	n/a	n/a	21 vs. 22	31
Göpel et al. (44)	NCPAP ± LISA (108) vs. nCPAP ± INSURE (112)	26–28	8 vs. 13	n/a	14 vs. 15	46
Kanmaz et al. (45)	NCPAP + LISA (100) vs. nCPAP INSURE (100)	<30	10 vs. 20*	16 vs. 13	34 vs. 45	40
Tapia et al. (46)	NCPAP + INSURE (131) vs. MV (125)	800–1,500 g	7 vs. 10	8 vs. 9	14 vs. 19	30

NCPAP, Nasal continuous positive pressure ventilation; IMV, Invasive mechanical ventilation; INSURE, INtubation, SURfactant and Extubation; LISA, Less invasive surfactant administration; GA, Gestational age (weeks) *P < 0.05.

## NIPPV in the Delivery Room

There are no randomized clinical trials of using NIPPV compared to CPAP in the DR. Infants are typically placed on either CPAP or NIPPV after initial resuscitation. In a retrospective study in very low birth weight infants, comparing positive pressure ventilation (PPV) using a face mask to directly placing on NIPPV with RAM nasal cannula at birth, NIPPV use was associated with a significantly decreased need for intubation in the DR (31 vs. 85%) including among the extremely preterm infants born at 24–27 weeks of gestation, decreased need for chest compressions (11 vs. 31%), and decreased the need for IMV at 24 h of age (38 vs. 66%) (28).

## Use of Sustained Lung Inflation in Delivery Room

Sustained lung inflation (SLI) strategy may result in better lung recruitment immediately after birth through delivery of a PIP of 15–30 cmH_2_O for a sustained period of time, typically, 10–15 s to the infant airways via a nasopharyngeal tube or mask or NC, followed by CPAP. SLI procedure creates a transepithelial pressure gradient across the alveolar-capillary membrane and helps to move fluid from the alveoli into the interstitial space and subsequent removal of this fluid via lung lymphatics and pulmonary microcirculation. SLI superimposed on PEEP may have beneficial effects, like, maintaining adequate FRC, promoting optimal gas exchange, improving lung mechanics, and reducing the need for intubation in the DR (50). A recent large multicenter study in extremely preterm infants requiring resuscitation at birth, a ventilation strategy involving 2 SLIs at maximal PIP of 25 cmH_2_O for 15 s, compared with standard intermittent positive pressure ventilation, did not reduce the risk of BPD or death at 36 weeks postmenstrual age. The study was stopped early due to safety concerns with increased rates of death in the infants receiving SLI (51). Reasons for increased mortality with SLI in this study are not clear. Updated systematic review and meta-analysis of SLI vs. intermittent positive pressure ventilation and continuous positive airway pressure for the prevention of hospital mortality and morbidity in preterm infants showed no difference in the risk of the primary outcome of death in the delivery room or before hospital discharge, although SLI was associated with increased risk of death in the first 2 days after birth, with no evidence of efficacy for SLI prevent other neonatal morbidities. Duration of mechanical ventilation was shorter in the SLI group but did not translate into better long term pulmonary outcomes. These findings do not support the routine use of SLI in preterm infants at birth in the delivery room (52, 53).

## High Flow Nasal Cannula for Stabilization in the Delivery Room

Only one study had evaluated HFNC during stabilization at birth. Reynolds et al. performed a pilot study evaluating use of HFNC (6–7 LPM) in stabilizing infants <30 weeks' gestation; 25 of 28 infants were successfully stabilized with HFNC, 48% of the infants received surfactant and 60% of the infants remained on HFNC at 72 h of age (54). They concluded that it is feasible to use HFNC in preterm infants. Additional studies in extremely preterm infants are needed.

## Early Use of NIV in NICU

The major risk factors for BPD in extremely infants are treatment with oxygen and IMV. A meta-analysis of 7 randomized controlled trials including 3,289 patients showed that avoiding IMV reduced the combined outcome of death or BPD in preterm infants <30 weeks' gestational age (47). At present, NCPAP and NIPPV are the two most common modes used either as a primary mode or rescue mode of NIV support in the NICU.

## Early NCPAP

Previous systematic reviews of randomized clinical trials of preterm infants found that the early use of NCPAP to avoid IMV decreased BPD, death, or both compared with the respiratory management using routine intubation (48, 55). However, one major disadvantage of using only NCPAP without intubation is a delay in the administering surfactant that is generally given via an endotracheal tube after intubation. A large multicenter study comparing early NCPAP to intubation and surfactant within 1 h of age in infants less than 28 weeks of gestation did not show any significant differences in long term morbidities (40). Several factors in extremely preterm infants including gestational age <26 weeks, birth weight <750 g, need PPV in the DR, FiO_2_ >0.30 and severe RDS on chest x-ray contribute to NCPAP failures (56). CPAP failure is associated with increased risk of mortality and major morbidities, including BPD, both in infants <29 weeks' and in infants between 29 and 32 weeks' GA (57).

## Use of NCPAP With Surfactant Therapy

Few RCTs have evaluated routine NCPAP with NCPAP after surfactant via INSURE or LISA technique (41–46). In the DR management trial, infants 26 to 29 weeks' gestation were randomized to 3 groups: prophylactic surfactant followed by a period of mechanical ventilation for at least 6 h, prophylactic INSURE within 30 min followed by bubble NCPAP or initial management with bubble NCPAP and selective surfactant treatment. There were no differences in death or moderate to severe BPD (NCPAP 4.1% vs. INSURE 7% vs. prophylactic surfactant 7.2%), and in pneumothorax (5.4% vs. 3.2% vs. 4.8%) in these 3 groups (41). In another multicenter, RCT from the South American Neocosur Network, early bubble CPAP and selective surfactant by INSURE technique reduced the need for mechanical ventilation and surfactant; however, there were no differences in the rates of death or BPD (46). A multicenter RCT from Germany included 220 infants between 26 and 29 weeks' gestation and reported decreased need for mechanical ventilation in infants treated with NCPAP and surfactant administration via LISA technique, but no decrease in BPD (44). However, a similar study from Turkey reported decrease in both the need for mechanical ventilation and BPD (10 vs. 20%) when treated with NCPAP and LISA (45). A recent study showed SurE technique using a thin catheter for surfactant delivery resulted in decreased need for MV and less BPD (11). In a meta-analysis including majority of the above mentioned studies, Fischer et al. concluded that avoiding early IMV by using NCPAP with or without surfactant resulted in a small but significant beneficial effect on preventing BPD, with a number needed to treat (NNT) of 35 (47) (Table 1). Furthermore, NCPAP failures in preterm infants <29 weeks GA is associated with increase in mortality, BPD, death or BPD, and necrotizing enterocolitis (NEC) (57).

## Early NCPAP vs. NIPPV Use in NICU

A major reason for lack of benefit in the NCPAP trials is due to high rates of NCPAP failures, requiring intubation within 3–7 days after randomization. Most common reasons for NCPAP failures are recurrent apnea, bradycardia or desaturation episodes, hypopnea, need for higher pressures (NCPAP > 8 cmH_2_O), and/or severe respiratory acidosis. NCPAP when used as a primary mode or following a period of IMV has been shown to result in failure rates of 31% to 83% (Table 1), requiring intubation or re-intubation. NIPPV augments NCPAP and has been shown to be more effective than NCPAP after extubation and in the treatment of apnea of prematurity (58, 59).

Several large RCTs comparing early NCPAP with early NIPPV have been published (60–69) (Table 2). Of the 10 studies reviewed, 6 studies enrolled patients prior to surfactant administration (60–64, 69), 2 studies had mixed enrollment (65, 66), and 2 studies enrolled after INSURE technique (67, 68). Four of these studies reported decreased rates of IMV (64, 65, 68, 69). Three studies reported decreased respiratory failure and duration of oxygen requirement (60, 61, 67). Three studies, where rescue surfactant via INSURE and LISA was provided, showed decreased rates of BPD (64, 68, 69). A recent Cochrane meta-analysis involving 10 trials enrolling 1,061 infants showed significantly decreased rates of respiratory failure [{relative risk}RR 0.61 (95% CI 0.51,0.82)], decreased need for intubation [RR: 0.78 (95% CI 0.64, 0.94)], and NNT to prevent one extubation failure was 17 with NIPPV. There were no differences in the rates of BPD (RR: 0.78; 95% CI 0.58, 1.06) and mortality (RR: 0.77 (95% CI 0.51, 1.15); however, in one study, combining surfactant with NIPPV led to a reduction in BPD. There were no differences in pneumothorax, NEC, IVH, and retinopathy of prematurity (70).

**Table 2:** Studies comparing NIPPV and NCPAP.

**References**	***n***	**Synchoniz-ation**	**Surfactant prior**	**GA, weeks**	**Intubation/respiratory failure**	**Death**	**BPD**
Armanian et al. (60)	98	No	No	<35	4 vs. 2	4 vs. 2	n/a
Bisceglia et al. (61)	88	No	No	28–34	2 vs. 2	0 vs. 0	4 vs. 8
Kirplani et al. (62)	185	Some	No	<30	21 vs. 29	3 vs. 4	19 vs. 14
Meneses et al. (63)	200	No	No	26–33	58 vs. 64	22 vs. 26	26 vs. 25
Kugelman et al. (64)	84	Yes	No	24–34	25 vs. 46^*^	0 vs. 0	2 vs. 17^*^
Sai Sunil Kishore et al. (65)	76	No	Some	28–34	19 vs. 41^*^	13 vs. 23	3 vs. 10
Salama et al. (66)	60	Yes	Some	28–34	10 vs. 20	0 vs. 3	3 vs. 6
Lista et al. (67)	40	Yes	Yes	28–34	10 vs. 15	0 vs. 0	0 vs. 0
Ramanathan et al. (68)	110	No	Yes	26–29	17 vs. 42^*^	2 vs. 2	22 vs. 39
Oncel et al. (69)	200	No	No	26–32	13 vs. 29^*^	4 vs. 6	7 vs. 16^*^

NIPPV, Nasal intermittent positive pressure ventilation; NCPAP, Nasal continuous positive pressure ventilation.

* *p < 0.05*.

One of the largest RCT involving 1,009 patients <1 kg at birth, comparing NCPAP with Si-PAP or NIPPV, reported no difference in extubation failures (61.8 vs. 59.5%), survival with BPD (31 vs. 33.9%), and death or BPD (36.7 vs. 38.4%) (62). In this pragmatic study, more than half of the centers used IFD device to deliver “NIPPV,” and in centers using a ventilator to deliver NIPPV, maximum PIP that could be used was limited to 18 cmH_2_O. Even though, the authors described this study as NIPPV vs. NCPAP, this was truly a study comparing Si-PAP with NCPAP. Post-randomization failures needing intubation were very high, most likely, secondary to lower delta pressures used in the “NIPPV” group. However, Lemyre et al. included 185 infants from this study who were randomized prior to intubation and surfactant administration in their Cochrane review and found decreased need for intubation and respiratory failure (70).

Early NIPPV appears to be superior to NCPAP alone for decreasing respiratory failure and the need for intubation and IMV among preterm infants with RDS (70). Another important factor is related to the devices used to deliver NIPPV. NNT to decrease respiratory failure and intubation with a ventilator delivered NIPPV was 13 (70). Current evidence suggests early NIPPV delivered with a ventilator and minimally invasive technique for early, rescue surfactant therapy, like, LISA may be the most effective strategy to minimize IMV and improve outcomes in extremely preterm infants. Recommended settings for NIPPV, NCPAP, and HFNC are shown in the Table 3.

**Table 3:** Suggested settings for CPAP, Bi-PAP, NIPPV, and HFNC.

**Mode of NIV**	**Initial settings**	**Max settings**	**Weaning parameters**	**Lowest settings**
NCPAP	5–6 cmH_2_O	8–10 cmH_2_O	1 cmH_2_O	4 cmH_2_O
Bi-PAP	High Pressure 10 cmH_2_O Low Pressure 5 cmH_2_O Rate 20/min	High Pressure 15 cmH_2_O Low Pressure 8 cmH_2_O Rate 30/min	1 cmH_2_O wean the rate by 2–4 /min every 6 h	High/Low Pressure 8/5 cm H_2_O Rate 0
NIPPV	PIP 20 cmH_2_O PEEP 6 cmH_2_O Inspiratory time 0.5 s Rate 40/min	PIP 35-38 cmH_2_O PEEP 8–10 cmH_2_O Rate 50/min	wean PIP first by 1–2 until lowest possible PIP wean the rate by 2–4 /min every 6 h	PIP 12 or 15 cmH_2_O PEEP 4–5 cmH_2_O Rate 20/min
HFNC gas flow	4–6 L/min	8 L/min.	0.5–1.0 L/min	1–4 L/min

*NIV, non-invasive ventilation; NCPAP, nasal continuous positive airway pressure; NIPPV, nasal intermittent positive pressure ventilation; HFNC, high flow nasal cannula; PIP, peak inspiratory pressure; PEEP, positive end expiratory pressure*.

There are no studies comparing weaning strategies from NIPPV to CPAP. Several factors are to be considered while weaning including underlying pulmonary disease, intermittent hypoxic episodes, post-natal age, growth, oxygen requirement, and gas exchange. Individual patient specific weaning strategy is encouraged. For extremely preterm infants we (HM, MB, RR) typically wean PIP first before weaning the rate. When the PIP is around 12 or 15 cmH_2_O, PEEP at 5–6 cmH_2_O, and FiO_2_ <030, we wean the rate by 2–4 bpm every 6 h and transition to NCPAP. When patient is stable on NCPAP for 12–24 h, we wean to low flow NC (<2 lpm).

## NIPPV vs. NCPAP Post-Extubation

Infant receiving invasive ventilation are at high risk for developing complications such as increased hemodynamic instability, increased airway resistance, acute and chronic airway trauma, increased ventilation associated infections and reduced clearance of secretions. Minimizing IMV and extubating to NIV may aid in avoiding these undesirable side effects. Choice of post-extubation respiratory support is based on several factors including level of respiratory support at the time of extubation, duration of respiratory support, underlying lung pathology, and associated clinical problems as well as infant's hemodynamic status.

The 2017 Cochrane meta-analysis compared NIPPV and NCPAP for respiratory support post-extubation and included 10 studies with 1,431 infants and reported decreased rates of respiratory failure [RR: 0.70 (95% CI 0.60, 0.80)] and reintubation rates [RR: 0.76 (95% CI 0.65, 0.88)] with NIPPV without increase in gastrointestinal side effects (59). NIPPV reduced the incidence of extubation failure and the need for re-intubation within 48 h to 1 week more effectively than NCPAP; however, it had no effect on BPD or mortality (59, 71–74). In a recent systematic review, Ferguson et al. concluded that NIPPV is superior to NCPAP in preventing extubation failure [(RR 0.70, 95% CI 0.60, 0.81; NNT 8; 95% CI 5, 13)] (Table 4) (75).

**Table 4:** Interventions to improve rates of successful extubation in preterm infants.

**Preventing extubation failures**	**Risk ratio [95% CI]**	**NNT [95% CI]**
NCPAP vs. Head-Box	0.59 [0.48–0.72]	6 [3–9]
NCPAP vs. nHF	1.11 [0.84–1.47]	–
Methylxanthines	0.48 [0.32–0.71]	4 [2–7]
DOXAPRAM	0.80 [0.22–2.97]	–
NIPPV vs. NCPAP	0.70 [0.60–0.81]	8 [5–13]
NS-NIPPV or Bi-PAP vs. NCPAP	064 [0.44–0.95]	8 [4–50]
_S_NIPPV vs. NCPAP	0.25 [0.15–041]	4 [2–5]
NS-NIPPV or sNIPPV vs. NCPAP	0.28 [0.18–043]	4 [2–5]

*NS-NIPPV, non-synchronized NIPPV; sNIPPV, synchronized NIPPV; nHF, High flow nasal cannula. Ferguson et al. (75)*.

## Synchronized NIPPV

A clinical report by the American Academy of Pediatrics concluded that synchronized NIPPV (sNIPPV) decreases the frequency of extubation failure but the evidence for non-sNIPPV or Bi-PAP is inconclusive (76). The main reason for the absence of evidence is directly attributed to the lack of approved devices to provide an effective synchronization during NIV in USA. The most studied system for synchronization during NIPPV in newborns is the Graseby capsule (77, 78), but this system is no longer available. At present, there are no devices in the United States that are capable of providing sNIPPV, except for neurally adjusted ventilatory assist (NAVA). However, there are devices available in other parts of the world where flow synchronization as well as Graseby capsule have been successfully used to provide sNIPPV (79).

## HFNC for Primary Respiratory Support in the NICU

In a recent international, multicenter, randomized, non-inferiority trial, 564 preterm infants with gestational age >28 weeks (HISPTER trial) were randomized to HFNC or NCPAP. When used as primary support in preterm infants with respiratory distress, HFNC use resulted in significantly higher rates of treatment failure than NCPAP (25.5 vs. 13.3%) (80). Systematic reviews including 2016 Cochrane review and a more recent systematic review reported CPAP was superior to HFNC in preventing treatment failure and intubation [RR 1.83 (95% CI 1.43, 2.35)] in favor of CPAP (81, 82). One pilot study of 76 infants <35 weeks GA and >1,000 g birth weight compared HFNC with NIPPV as a primary mode of respiratory support and found no difference in rate of intubation and MV; however, HFNC was associated with longer duration of oxygen support (83).

## HFNC vs. NCPAP in the NICU

In one of the largest retrospective study of 2,487 extremely preterm infants, Taha et al. reported that HFNC use was associated with higher risk of death or BPD and longer length of stay when compared to NCPAP (84). The 2016 Cochrane review of HFNC compared with NCPAP to prevent extubation failure included six trials (934 infants) and found no difference in the rate of treatment failure [RR 1.21 (95% CI 0.95, 1.55)] or reintubation RR: 0.91 (95% CI 0.68, 1.20)] within 7 days, but reported a lower rate of nasal trauma [(RR 0.64 (95% CI 0.51, 0.79)] (81). A more recent systematic review included 3 more trials and found similarly no difference in rate of treatment failure [RR 1.21 (95% CI 0.97, 1.50)] and intubation rate [RR 0.98 (95% CI 0.77, 1.24)]. However, majority of the studies included infants greater than 28 weeks' gestation and currently there is insufficient evidence to support use of HFNC as primary mode or for post-extubation respiratory support in infants less than 28 weeks' gestation (82).

## Non-Invasive Neurally Adjusted Ventilatory Assist (NIV-NAVA)

### Mechanism

NAVA is a newer mode of ventilation that utilizes electrical activity of diaphragm (Edi) using a special nasogastric tube embedded with electrodes to provide synchronized breaths (85). An electrical signal is generated in the respiratory center in the brainstem and travels via the phrenic nerve to stimulate the diaphragm. The Edi catheter with electrodes is inserted and adjusted in the esophagus to provide an optimal signal from diaphragm. Edi mas as well as Edi min values are detected by the electrodes and transmitted to the ventilator. The ventilator assists the spontaneous breath by delivering a proportional pressure as determined by NAVA level. The PIP delivered is proportional to the amount of Edi. Systematically increasing NAVA levels increases PIP while maintaining Edi until the breakpoint is reached. Further increases in NAVA leads to decrease in Edi. This breakpoint is increased after extubation in premature infants (86). The initiation, duration, size, and termination of breath are controlled by the patient, and thus, potentially offering full synchronization (85). Typical settings of NIV NAVA are shown in the Table 5. NAVA levels are typically adjusted to keep Edi peak goal of 5–15 5 μV and Edi min is kept usually between 2 and 4 5 μV. If the Peak Edi is too high, NAVA level is increased to reduce the patient's work of breathing whereas if the Peak Edi is too low, the NAVA level is reduced and weaning considered. If Edi min is too high, then additional PEEP is provided whereas for low Edi min PEEP is reduced. Weaning the patient is considered after a decline in the Edi signal and peak pressure essentially showing improvement in diaphragm performance. Detailed guide for initial set up of NIV-NAVA can be accessed at https://www.neonatologytoday.net/newsletters/nt-apr12.pdf.

**Table 5:** NIV NAVA suggested settings.

	**Initial settings**	**Maximum**	**Wean**	**Minimum**
NAVA level Edi max between 5–15 μV	2 cm H_2_O/μV	4 cm H_2_O/μV	0.2 to 0.5 cmH_2_O/μV For Edi max >15 μV	0.5 cmH_2_O/μV
PEEP	6 cmH_2_O	8-10 cmH_2_O	1 cmH_2_O	5 cmH_2_O
Edi Trigger	0.5 μV	2 μV	Adjust as needed	
Backup Pressure Control above PEEP	15 cmH_2_O	30–35 cmH_2_O	1–2 cmH_2_O	Per NIPPV
Rate	40 /min	Per NIPPV	Per NIPPV	Per NIPPV
Inspiratory time	0.5 s	–	–	–
Trigger sensitivity	1 to 2	–	–	–

*Edi, electrical activity of diaphragm; PEEP, positive end expiratory pressure; NIPPV, nasal intermittent positive pressure ventilation*.

### Use of NIV NAVA in NICU

Stein et al. in a retrospective study reported that in preterm infants managed on NAVA mode maintained better blood gases with lower PIP and oxygen requirements compared to synchronized IMV plus pressure support (SIMV+PS) mode of ventilation (87). Lee et al. in a randomized crossover study also reported that NAVA lowered PIP and reduced respiratory muscle load in preterm infants when compared to SIMV+PS (88). Kallio et al. performed a randomized controlled trial in 60 infants between 28 and 36 weeks GA comparing NAVA and conventional ventilation and found no difference in duration of invasive ventilation (89). Studies using NIV-NAVA mode in extremely preterm infants are limited with some of the recent studies showing promising results (Table 6) (16, 90–95). Larger trials are needed to determine if NIV-NAVA is a better mode to provide sNIPPV to prevent BPD.

**Table 6:** Studies comparing Noninvasive neutrally adjusted ventilator assist (NIV NAVA) to other forms of non-invasive ventilation.

**References**	**Type of study**	**Comparison**	**GA (*n*)**	**Reintubation**	**CO_**2**_ clearance**	**Syn**	**Complications**	**Oxygen requirement/IMV duration**	**Outcome** **(Death/BPD)**
Lee et al. (90)	Retrospective	NCPAP	<30 (30)	*P* = 0.04	NS	–	–	NS	NS
Kallio et al. (91)	Prospective	NCPAP	28–36 (40)	NS	NS	NS	NS	NS	NS
Yonehara et al. (92)	Retrospective	NIPPV	<30 (34)	NS	–	–	NS	–	–
Lee et al. (93)	Observational crossover	NIV-PS	<32 (15)	–	–	*P* <0.001	–	–	–
Gibu et al. (16)	Observational crossover	NIMV	<37 (11)	–	NS	*P* < 0.01	*P* < 0.01	*P* < 0.01	–
Yagui et al. (94)	Randomized controlled	NCPAP	Preterm <1,500 g (123)	NS	–	–	NS	*P* < 0.01	NS
Yagui et al. (95)	Retrospective	NCPAP	ELBW	*P* = 0.02	–	–	NS	*P* = 0.02	NS

NIPPV, Nasal intermittent positive pressure ventilation; NCPAP, Nasal continuous positive pressure ventilation; NIV-PS, Non-invasive ventilation-Pressure support; GA, Gestational age.

*IMV: invasive mechanical ventilation; Syn-synchronization*.

## Nasal High Frequency Ventilation (NHFV)

To decrease the need for intubation and improve ventilation in infants with hypercarbia, using NHFV modalities such as nasal high frequency flow interrupter (NHFFI), nasal high frequency oscillatory ventilator (NHFOV) (96), nasal high frequency percussive ventilator (NHFPV) or nasal high frequency jet ventilator (NHFJV) using standard nasal interfaces have been reported. Three variables that impact the delivery of tidal volume are inspiratory time (IT), amplitude, and frequency. Longer IT, higher amplitude, and lower frequency are associated with larger tidal volume delivery during NHFV. Addition of NIPPV breaths during NHFJV also improves ventilation (97). NHFV using HFFI device, Infant Star was first reported in 1998. In this observational study of 21 preterm infants, significant improvement in ventilation was seen after starting NHFFI (98). Another study describing successful use of NHFFI in 14 patients was reported in 2008 (99).

## Use of NHFV in NICU

In a randomized, controlled trial comparing NHFPV with NCPAP in 40 term neonates delivered by cesarean section, with a diagnosis of transient tachypnea of the newborn (TTN), NHFPV was well-tolerated and more effective in improving oxygenation when compared with NCPAP (100). Using a nasopharyngeal tube to deliver NHFOV in 20 preterm neonates during weaning from IMV, NHFOV was successfully used in 91% of the patients at first attempt at extubation (101). Mukerji et al. reviewed 52 patients treated with rescue NHFOV when other NIV modes failed. Intubation was avoided in 58% of the cases (102). In a small randomized, controlled trial involving 39 patients with a birth weight <1,250 g, NHFOV was found to be not superior to Si-PAP (103). Most likely reason for lack of success in this pilot study was the use of lower MAP in the NHFOV group. In a recent meta-analysis of 8 RCTs involving 463 patients, NHFOV significantly improved CO_2_ clearance and reduced the need for intubation compared with NCPAP/bi-phasic CPAP (104). There are no clinical trials using NHFJV. There is only one reported case series showing successful use of NHFJV in selected extremely preterm infants immediately after extubation from IMV (105). In a review of 6 NHFV studies involving 111 patients; different inspiratory time, amplitude, and frequency were used (96). Suggested settings for NHFV are shown in Table 7.

**Table 7:** Suggested settings for Nasal High Frequency Ventilation.

Frequency, Hz	Start at 6–8 Hz; May decrease to 4 Hz in patients with hypercapnia; If using HFJV, start at 300 bpm (5 Hz) and may decrease to 240 bpm (4 Hz)
Amplitude, cmH_2_O	MAPx2; Start at 20–30 cmH_2_O; May increase to as high 70 cmH_2_O. If using during weaning, set Amplitude equaling PIP prior to extubation
I: E ratio	Start at 1:1; May change to 1:2 in cases of gas trapping; If using HFJV, jet valve on time: 20 ms and may increase to 30–34 ms to improve oxygenation and increase tidal volume delivery
Mean Airway Pressure (MAP), cmH_2_O	MAP: Start with the same MAP as on SIMV or 2–3 cmH_2_O higher than CPAP; Start at 8–10 cmH_2_O; May increase as needed based on FiO_2_ and or lung expansion
NIPPV Back up rate	If available, use rates between 30 and 40 bpm; If using HFJV, keep the NIPPV settings same as before adding NHFJV

*I:E ratio, Inspiration: Expiration ratio; HFJV, high frequency jet ventilator; NIPPV, nasal intermittent positive pressure ventilation; MAP, Mean Airway Pressure; SIMV, Synchronized intermittent mandatory ventilation*.

## Complications of Using NIV

Even if NIV offers a number of benefits over IMV some extremely preterm infants may develop complications while receiving NIV. Majority of complications are related to injury to nasal mucosa as well as nasal septum.

## Nasal Injuries During NIV

One of the major problems with NIV use in the NICU in the occurrence of septal or nasal mucosal injuries resulting in nasal deformities. Snugly fit nasal prongs may put extremely preterm infants at risk for causing nasal trauma including erythema or blanching, ulceration, and columellar necrosis. Reported incidence of these complications varies from 20 to 60% in neonates. Both frequency and severity of nasal trauma has been shown to be higher in infants at lower gestational age (>90% in neonates <28 weeks of gestational age), lower birth weight, longer duration of NCPAP and longer NICU stay (106). It is important to choose the right interface with correct size of prongs as well as fittings as onset of nasal injury to the columella has been reported to occur within a mean of 2–3 days of CPAP commencement, and in some cases occurring as early as 18 h after commencement. The use of nasal barrier dressings and nasal masks as an alternative to binasal prongs may be effective interventions to reduce nasal injury. HFNC causes less nasal injury than CPAP, but it may not provide sufficient respiratory support for the smallest, sickest preterm infants (107). These complications not only have cosmetic or functional sequelae but also place the infants at risk for developing nosocomial infections. In one study nasal breakdown with the INCA prongs and subsequent use of the RAM NC did not worsen or contribute nasal injuries. Also, there were no new instances of nasal breakdown or injury reported with use of the RAM NC (108). A recent study showed skin or mucosal breakdown with RAM NC was significantly lower compared to other nasal interfaces (8 vs. 53%, *P* < 0.001) (109). Common recommendations for prevention of nasal trauma due to NCPAP in neonates include careful monitoring of the nose, avoidance of pressure, friction, and moisture.

## Other Complications With NIV

Systemic complications related to NIV use are usually rare and account for less than 5% of patients. Pneumothorax can occur in acute phase and it is most commonly related to underlying lung disease rather than NIV itself. Also, after surfactant administration with sudden change in the compliance may lead to air leaks as well. HFNC may have reduced occurrence of pneumothorax compared to CPAP. Small pnuemothoraces usually resolves spontaneously, and one may not need to change the modality if infant is otherwise stable. Rarely an intervention is needed to evacuate the air to re-inflate the affected lung. A recent study showed a decrease risk of pneumothoraces after implementing NIV in the delivery room instead of intubation in extremely low birth weight infants (3).

It is common to observe abdominal distension with or without feeding intolerance with NIV. Some infants may need to have orogastric tube to vent the stomach and to evacuate air. There may be transient feeding intolerance. Decrease in flow may help relieve gastric distension as well. Infants who have undergone upper gastrointestinal surgery are at higher risk of complications, such as leak at the site of anastomosis if NIV is used in the immediate post-operative period (110). In a small case series, Pandita et al. reported facial palsy in 3 patients, who were on NCPAP and speculated that pressure over the stylomastoid formen by the NCPAP interface might have contributed to ipsilateral facial palsy (111).

## Conclusions

In extremely preterm infants, optimal pulmonary outcomes could be achieved by minimizing the duration of IMV. NIV is currently best provided by early use of NIPPV from DR through 32 to 33 weeks postmenstrual age in the NICU. NCPAP may be used when weaning from NIPPV, followed by low flow nasal cannula (<2 LPM) in extremely preterm infants to minimize lung injury. sNIPPV could be delivered using NIV NAVA but needs more evidence to support its use in this specific population. NHFV using NHFOV as well as NHFJV have the potential as a rescue mode for use in this population especially when the lung disease is severe requiring higher pressure (PIP >30 cmH_2_O) to improve gas exchange.

## Author Contributions

YS: Drafting of the initial manuscript, critical revision of the manuscript for important intellectual content. RR: Review of the literature, creation of tables, checking and adding references, creation of figures, drafting and subsequent revisions of the manuscript for important intellectual content. MB: Review of the literature, creation of tables, checking and adding references, drafting and subsequent revisions of the manuscript for important intellectual content. HM: Review of the literature, creation of tables, drafting and subsequent revisions of the manuscript for important intellectual content. All Authors reviewed the final version of this manuscript and approved for submission.

## Conflict of Interest

RR has a joint patent on RAM Nasal Cannula and receives royalty payments. The remaining authors declare that the research was conducted in the absence of any commercial or financial relationships that could be construed as a potential conflict of interest.
